# Senescence utilises inflammatory caspases to drive SASP

**DOI:** 10.18632/aging.102031

**Published:** 2019-06-17

**Authors:** Kimberley A. Wiggins, Murray CH. Clarke

**Affiliations:** 1 Division of Cardiovascular Medicine, Department of Medicine, University of Cambridge, Addenbrooke’s Hospital, Cambridge, CB2 0QQ, UK

**Keywords:** cellular senescence, inflammasomes, caspase-5, cytokines, aging

Senescence is a state of stable cell cycle arrest that occurs in response to cellular stress and prevents transmission of defects to the next generation. Cellular senescence is an important protective process with roles in development, tissue homeostasis, and wound healing. However, senescence is also induced by pathophysiological stimuli, including ROS, oncogene activation, cytotoxic drugs and aging [1]. Senescent cells are implicated in multiple diseases including cancer, arthritis, atherosclerosis and a diminished healthspan during aging.

The senescence-associated secretory phenotype (SASP) is an important hallmark of senescence that contributes to normal physiology and disease. The SASP is characterised by the release of inflammatory cytokines, chemokines, growth factors and proteases. This reinforces senescence through autocrine and paracrine signalling, and recruits and instructs immune cells to clear senescent cells. However, senescent cells can also generate an inflammatory environment. Thus, the SASP is often considered a double-edge sword. Whilst promoting immune-mediated clearance of pre-malignant senescent cells is a powerful barrier against transformation, the SASP from uncleared senescent cells, or those arising during natural aging, can create an inflammatory milieu permissive to disease.

The SASP is regulated by interleukin-1 alpha (IL-1α) [2], which is synthesised as a pro-protein that requires proteolytic cleavage for full cytokine activity [3–5]. IL-1α, and its ‘sibling’ IL-1β, bind the type-1 IL-1 receptor (IL-1R1) to induce cytokine expression, adhesion and MHC/costimulatory molecule expression, Th17 cell differentiation, and T-cell expansion and survival. These potent actions are regulated by autoinhibition of the cytokine proforms, a receptor antagonist (IL-1RA) and a decoy receptor (IL-1R2) [6]. In addition to its constitutive expression in many cell types, pro-IL-1α also associates with the cell surface. This membrane-bound form of IL-1α is suggested to regulate the SASP, but the mechanism of IL-1α activation during senescence is unknown.

Previous studies have suggested that NLRP3 inflammasomes modulate the SASP [7], even though caspase-1 cannot activate IL-1α. However, our recent research has demonstrated that caspase-5, which lies upstream of NLRP3 in the non-canonical inflammasome pathway, induces IL-1α activity and regulates the SASP during oncogene-induced senescence (OIS) *in vitro* and *in vivo* [5]. The non-canonical inflammasome is predominantly studied in the context of bacterial infection where cytosolic LPS activates human caspase-4/5, or the murine orthologue caspase-11, which cleaves the pore-forming protein gasdermin D to induce pyroptosis and NLRP3 inflammasome activation [6]. Recent research also implicates the non-canonical inflammasome in sterile inflammation, of which the SASP is an important yet rarely cited example.

Our recent investigation demonstrated that caspase-5 or -11, but not caspase-4 or -1, specifically cleaves human or mouse pro-IL-1α at a highly conserved site. We demonstrated that caspase-5/11 is required for IL-1α release from cells, in response to both intracellular LPS in macrophages and H-RAS-induced senescence in fibroblasts. siRNA-mediated caspase-5 knockdown reduced levels of cell-surface and secreted IL-1α, and impaired release of the common SASP factors IL-6, IL-8 and MCP-1 from senescent IMR-90 and WI-38 fibroblasts. Importantly, although pro‐IL‐1β was upregulated in our model of OIS, negligible amounts were proteolytically matured or secreted, and the SASP was not IL-1β-dependent. The relevance of this pathway was also demonstrated *in vivo,* using the well-established model of hepatocyte senescence that uses hydrodynamic tail vein injection to deliver bicistronic constructs containing *Nras* and shRNAs, which undergo transposon‐mediated stable integration and induce OIS. We observed upregulation of caspase-11 in NRAS+ senescent hepatocytes, and found that *Casp11* knockdown caused accumulation of senescent cells over time concomitant with reduced infiltrating macrophages and immune cell clusters - supporting a clear role for caspase-11 in immune-mediated senescent cell clearance *in vivo*.

Our work identifying caspase-5 as a novel regulator of IL-1α activity and the SASP raises several important questions for future research. Firstly, it will be important to understand how caspase-5 is activated in senescent cells. We demonstrated that knockdown of *CGAS* results in reduced caspase-5 expression and an impaired SASP, and hypothesised that cGAS/STING activated by cytosolic chromatin in senescent cells may drive *CASP5* expression via type I interferons. However, more experiments are required to validate this pathway in SASP regulation *in vitro* and *in vivo.* In addition, our experiments are limited to OIS, and it would be important to determine if caspase-5 mediates IL-1α activation and the SASP during developmental, replicative or DNA damage-induced senescence.

The discovery of caspase-5 as a novel regulator of IL-1α in sterile and non-sterile inflammation has several important clinical implications. Targeting caspase-5 may be a therapeutic strategy that leaves canonical immune responses via caspase-1 and -4 intact. For instance, radiotherapy and chemotherapy induce DNA damage that can trigger tumour cell senescence. However, these non-selective therapies also induce senescence in the underlying stroma, with IL-6 from senescent fibroblasts shown to be a reprogramming factor that drives pluripotency and proliferation of cancer stem cells surviving treatment [8]. Therefore, caspase-5 inhibition during treatment could lessen the chance of tumour recurrence. In contrast, because the SASP is IL-1α-dependent, the growing clinical use of IL-1 blockers such as Anakinra (IL-1RA) or IL-1α monoclonals for autoimmune or autoinflammatory conditions could potentiate the risk of transformation, due to SASP inhibition preventing clearance of pre-malignant senescent cells.

Cellular senescence and the SASP are a vital physiological response that maintains homeostasis at the cellular, tissue and organismal level. However, maladaptation of this programme, like any other, can have negative effects on host fitness. Early in life, senescent surveillance is vital to prevent transformation, whilst senescent cells accumulated during aging likely drive persistent low-level inflammation. Thus, understanding the molecular basis of senescence is vital to understand human disease.
